# Ethylenediamine pretreatment changes cellulose allomorph and lignin structure of lignocellulose at ambient pressure

**DOI:** 10.1186/s13068-015-0359-z

**Published:** 2015-10-29

**Authors:** Lei Qin, Wen-Chao Li, Jia-Qing Zhu, Jing-Nan Liang, Bing-Zhi Li, Ying-Jin Yuan

**Affiliations:** Key Laboratory of Systems Bioengineering (Ministry of Education), Tianjin University, Weijin Road 92, Nankai District, Tianjin, 300072 People’s Republic of China; SynBio Research Platform, Collaborative Innovation Center of Chemical Science and Engineering (Tianjin), School of Chemical Engineering and Technology, Tianjin University, Weijin Road 92, Nankai District, Tianjin 300072 People’s Republic of China; Institute of Microbiology Chinese Academy of Sciences, No.1 West Beichen Road, Chaoyang District, Beijing, 100101 People’s Republic of China

**Keywords:** Biomass, Pretreatment, Cellulose, Hydrolysis, Lignin, Ethylenediamine

## Abstract

**Background:**

Pretreatment of lignocellulosic biomass is essential to increase the cellulase accessibility for bioconversion of lignocelluloses by breaking down the biomass recalcitrance. In this work, a novel pretreatment method using ethylenediamine (EDA) was presented as a simple process to achieve high enzymatic digestibility of corn stover (CS) by heating the biomass–EDA mixture with high solid-to-liquid ratio at ambient pressure. The effect of EDA pretreatment on lignocellulose was further studied.

**Results:**

High enzymatic digestibility of CS was achieved at broad pretreatment temperature range (40–180 °C) during EDA pretreatment. Herein, X-ray diffractogram analysis indicated that cellulose I changed to cellulose III and amorphous cellulose after EDA pretreatment, and cellulose III content increased along with the decrease of drying temperature and the increase of EDA loading. Lignin degradation was also affected by drying temperature and EDA loading. Images from scanning electron microscope and transmission electron microscope indicated that lignin coalesced and deposited on the biomass surface during EDA pretreatment, which led to the delamination of cell wall. HSQC NMR analysis showed that ester bonds of *p*-coumarate and ferulate units in lignin were partially ammonolyzed and ether bonds linking the phenolic monomers were broken during pretreatment. In addition, EDA-pretreated CS exhibited good fermentability for simultaneous saccharification and co-fermentation process.

**Conclusions:**

EDA pretreatment improves the enzymatic digestibility of lignocellulosic biomass significantly, and the improvement was caused by the transformation of cellulose allomorph, lignin degradation and relocalization in EDA pretreatment.

**Electronic supplementary material:**

The online version of this article (doi:10.1186/s13068-015-0359-z) contains supplementary material, which is available to authorized users.

## Background

Lignocellulosic biomass is an abundant resource to produce fermentable sugars and consequent ethanol or other chemical products. The transformation of lignocellulose is beneficial to sustainable energy and environment. Cellulose and hemicellulose, accounting to more than half of total mass in lignocellulose, can be hydrolyzed to fermentable hexose (glucose) and pentose (xylose and arabinose) by specific enzymes, respectively [1]. However, the enzymatic digestibility of lignocellulose is low due to the structural recalcitrance [2]. Pretreatment is aimed to enhance cellulose conversion by deconstructing biomass structure and thus increasing the accessibility of enzymes and water to the components [3].

Alkaline pretreatments increases enzyme accessibility by degrading lignin and breaking linkage of lignin–carbohydrates [4], in which ammonia pretreatment is widely applied due to the recoverability of ammonia. Ammonia pretreatment employed either aqueous ammonia or liquid ammonia (ammonia fiber expansion, AFEX) [5–7]. Other amines have also been considered effective in pretreatment, such as *N*-methylmorpholine-*N*-oxide [8], *n*-butylamine and ethylenediamine (EDA) [9]. EDA/salts system was used to dissolve cellulose [10, 11]. EDA was also coupled with ionic liquid or organic solvent to remove lignin [12]. Besides, EDA has been widely used in changing cellulose allomorph as well as ammonia. Cellulose I_α_ and cellulose I_β_ are the predominant allomorphic forms of cellulose found in microbial and plants, respectively. EDA can penetrate the natural crystalline cellulose, break the hydrogen bonds between adjacent cellulose chains and form new hydrogen bonds between EDA and cellulose, and then convert cellulose I to EDA–cellulose I complex [13–16]. Upon removing EDA by washing with polar/non-aqueous solvents (e.g., ethanol) or drying under vacuum, the hydrogen bonds reform between cellulose molecules, and the crystal form of cellulose converts to cellulose III_I_ [17]. Cellulose III_I_ can also convert to cellulose I by heating above 200 °C [18]. It was reported that the enzymatic saccharification rate of cellulose III_I_ was much higher than cellulose I [19].

In order to achieve high cellulose conversion, most thermochemical pretreatments were carried out at relative high reaction severity, which requires high operating pressures, high temperatures and low biomass concentrations. As the thermal energy consumption in pretreatments almost linearly depends on solid-to-liquid ratio [20], the solid-state pretreatment (with high solid-to-liquid ratio) becomes much preferable and promising to improve energy efficiency and reduce water consumption [21, 22].

In this study, we developed a new pretreatment method using EDA with a simple process and high solid loading. We explored the pretreatment conditions to improve cellulose conversion, and investigated the mechanism of EDA pretreatment of lignocellulosic biomass. The fundamental insights from this report would be constructive to improve efficiency of lignocellulose hydrolysis.

## Results

### Dry method was crucial to increase EDA pretreatment efficiency

**Fig. 1:**
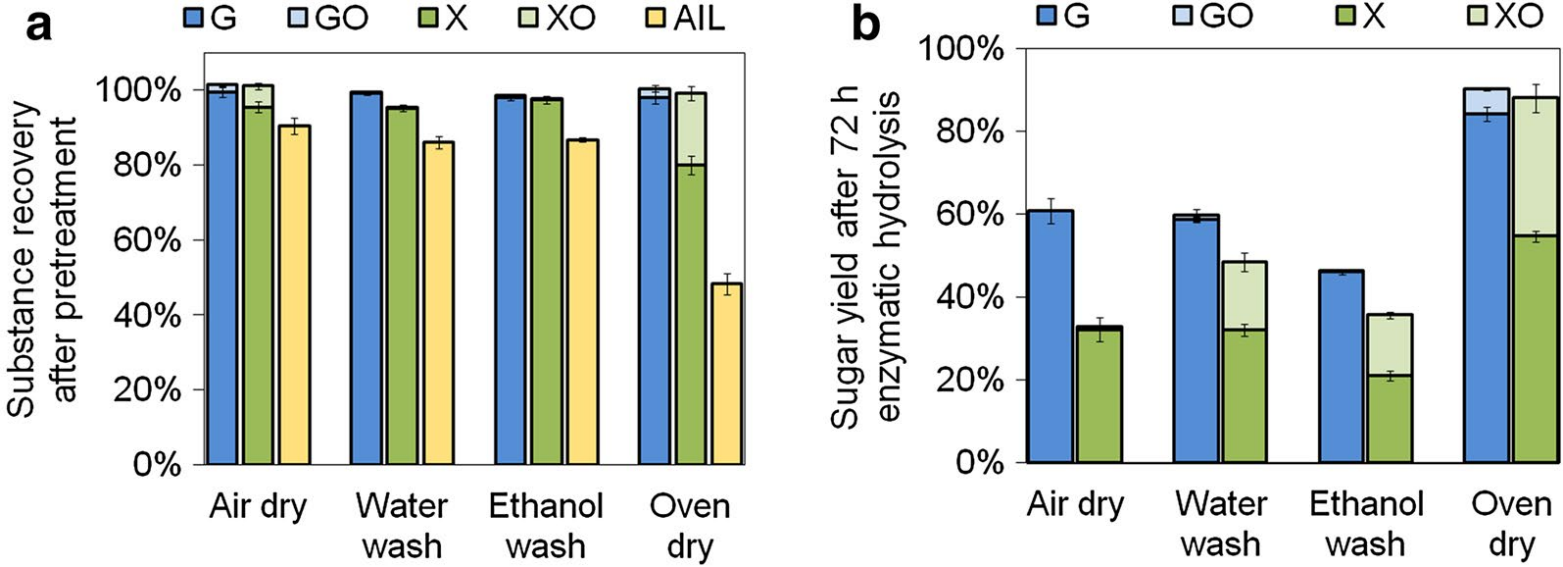
Effect of EDA separation methods on substance recoveries of CS after pretreatment (**a**) and sugar yields after following enzymatic hydrolysis (**b**). EDA loading was 1.0 mL/g biomass. EDA was separated from biomass with different methods before uniform mixing for 20 min at 20 °C. Air-drying was carried out at 20 °C in fume hood for 120 h. Water-washing and ethanol-washing were carried out by washing with water and ethanol, respectively, with a solid-to-liquid ratio of 1:10 (g:mL) for 3 times to remove EDA, followed by air-drying at room temperature in fume hood. Oven drying was carried out at 120 °C in oven for 30 min. *G* glucan, *GO* gluco-oligmer, *X* xylan, *XO* xylo-oligomer, *AIL* acid-insoluble lignin

Cellulose allomorphs after the separation of EDA and cellulose were affected by the dry methods [17]. Therefore, different dry methods (air dry, water wash, ethanol wash, and oven dry) were investigated to separate EDA from biomass after 20-min mixing of CS and EDA (1 mL EDA per g CS) at room temperature. The substrate recoveries after pretreatment and sugar yields after enzymatic hydrolysis were measured (Fig. 1, Additional file 1: Table S1). Glucan recoveries were above 98 % after all the separation methods, which is different from the EDA/salts dissolution effect of cellulose [10, 11]. Xylan recoveries were all above 95 % except for oven-dried CS, in which 19 % xylan degraded to soluble xylo-oligomer. Acid-insoluble lignin (AIL) recovery of oven-dried CS was 48 % and obviously lower than other drying methods (Fig. 1a) implied that xylan and AIL degradation was promoted at high temperature. Glucose and xylose yields of pretreated CS were all higher than the untreated CS (23 % for glucose and 21 % for xylose) after 72 h enzymatic hydrolysis (Fig. 1b). Oven-dried CS exhibited higher glucose yield (84 %) and xylose yield (55 %) than other drying methods. The higher sugar yields were consistent with the higher degradation of xylan and AIL.

**Fig. 2:**
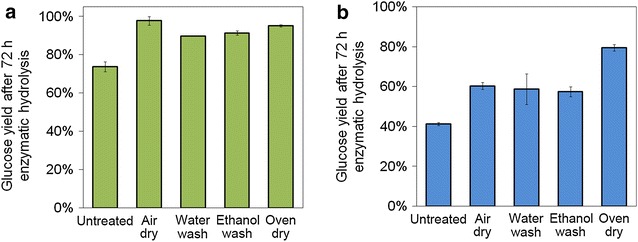
Effect of EDA separation methods on glucose yield after 72 h enzymatic hydrolysis using different substrates: **a** corn stover cellulose (CSC), **b** hemicellulose-free corn stover (HFCS)

To clarify whether the xylan and AIL content influenced sugar yields, two different substrates, hemicellulose-free corn stover (HFCS) and corn stover cellulose (CSC) were used to repeat the drying experiments (Fig. 2). CSC was prepared by acid hydrolysis to remove hemicellulose followed by chlorite acid bleaching to remove lignin. Glucose yield after 72-h hydrolysis of oven-dried HFCS increased 32, 35, and 92 % with respect to air-dried, water-washed, and untreated HFCS, respectively, which was consistent with the pattern of CS as substrate. However, digestibility of oven-dried CSC was comparable to air-dried and water-washed CSC. Therefore, we speculated that lignin played important role in enzymatic digestibility improvement in EDA pretreatment. Without lignin in cellulose substrate (i.e., CSC), glucose yields were similar among various drying methods. However, when lignin was not removed in substrate (i.e., HFCS and CS), the lignin content significantly affected the glucose yield.

### Pretreatment temperature and EDA loading impact sugars yield

**Fig. 3:**
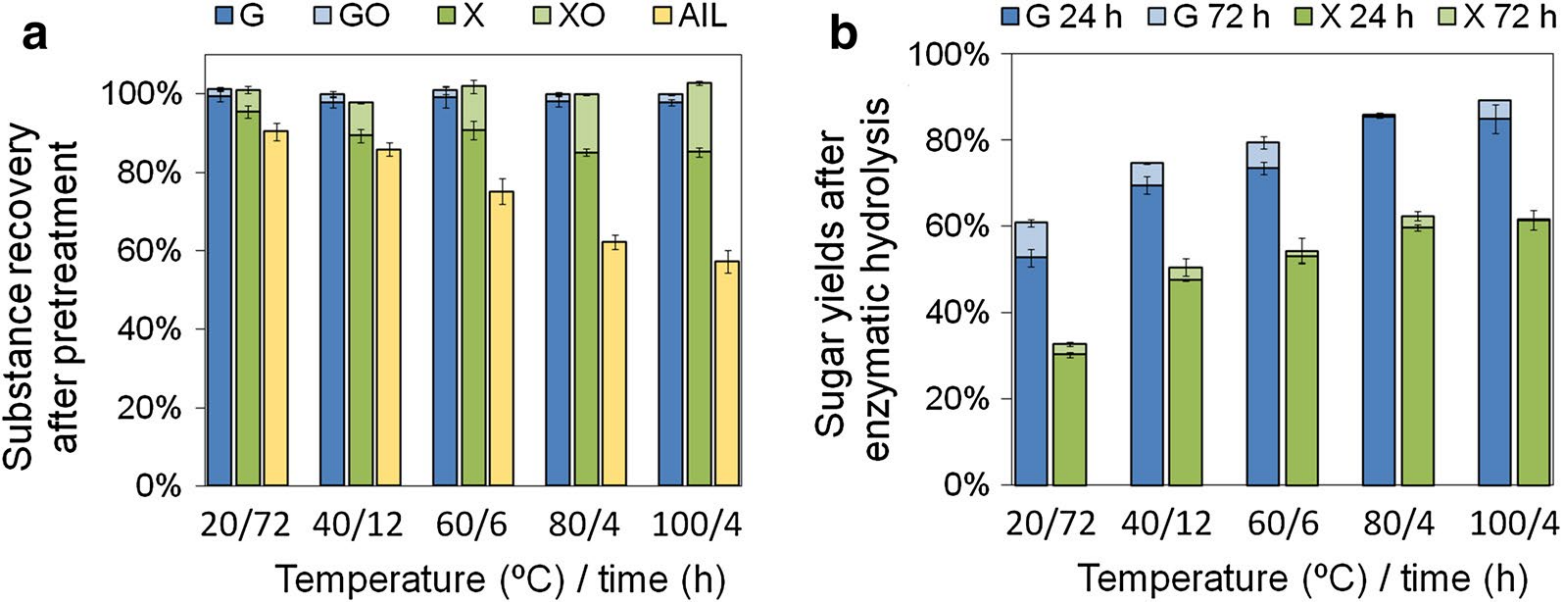
Effect of drying temperatures and times on substance recoveries in the pretreatment (**a**) and sugar yields in the following enzymatic hydrolysis (**b**). EDA loading was 1.0 mL/g biomass. The mixture was held at designated temperature in aluminum foil covered plate for 20 min followed by open drying at each condition. *G* glucan, *GO* gluco-oligmer, *X* xylan, *XO* xylo-oligomer, *AIL* acid-insoluble lignin

The effect of temperature on the substance recovery and enzymatic hydrolysis was explored. We standardized the EDA pretreatment into two processes: (1) reaction process of EDA and biomass in closed container; (2) drying process on open plate. The reaction process (20 min in this study) ensured the adequate contact of EDA with CS, while the drying process ensured removal of EDA from CS. Several temperatures below the EDA boiling point were tested in the drying process (Fig. 3). Drying times were varied at different temperatures in order to remove EDA from pretreated solids as much as possible (Fig. 3). Along with the increase of pretreatment temperature from 20 to 100 °C, AIL recovery successively decreased from 90 to 57 %, and conversion of xylan to xylo-oligomer increased from 6 to 18 %. Subsequent sugar yield after 72-h hydrolysis successively increased (glucose yield from 61 to 89 % and xylose yield from 33 to 62 %) and glucose yield exceeded 80 % beyond 60 °C. This result indicated that EDA pretreatment was effective to enhance cellulose digestibility at moderate temperatures. It was noticeable that solid recoveries after pretreatment were 110–120 % under these drying temperatures, indicating an evident part of EDA remained in pretreated solid (control trials of pretreatment at the same conditions without EDA addition showed 100 % solid recoveries).

**Fig. 4:**
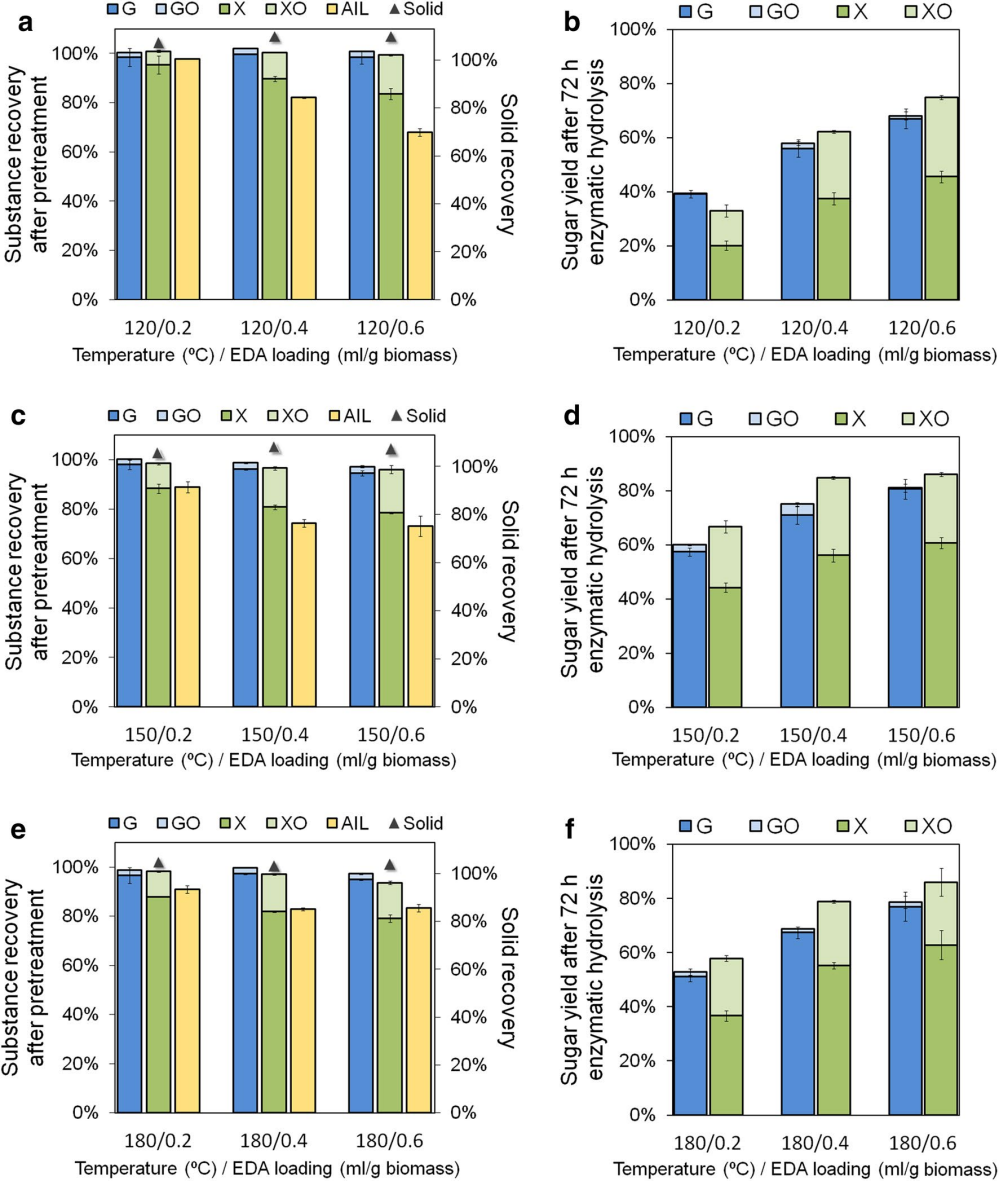
Effect of pretreatment temperatures and EDA loadings on substance recoveries after pretreatments (**a**, **c**, **e**) and sugar yields in the following enzymatic hydrolysis (**b**, **d**, **f**). The mixture was held at designated temperature in aluminum foil covered plate for 20 min followed by drying in open plate for 20 min at designated temperature

In order to reduce the residence of EDA and shorten the drying process, the effect of higher dry temperatures above EDA boiling point (120–180 °C) and lower EDA loadings (0.2–0.6 mL/g biomass) was investigated (Fig. 4, Additional file 1: Table S2). Reducing EDA loading showed great adverse effect on sugars yield. When EDA loading was reduced from 1.0 to 0.6 and to 0.2 mL/g biomass, glucose yield at 72-h hydrolysis after drying at 120 °C decreased from 89 to 67 % and to 39 %, respectively (Figs. 1b, 4b). The decrease of sugar yield along with the EDA loading reduction was also observed for the biomass drying at 150 °C and 180 °C. This result revealed that EDA loading is the key factor for the pretreatment and it was inadequate until 1.0 mL/g biomass. In contrast to low temperatures, AIL recovery increased from 120 to 180 °C, perhaps because the lignin degraded less efficiently during rapid gasification of EDA at higher temperatures. Solid recovery after pretreatment decreased as the temperature increased (109, 105, and 102 % with respect to 120, 150, and 180 °C at EDA loading of 0.6 mL/g biomass, respectively), reflecting EDA residue in the solids reduced.

### Cellulose allomorphs

**Fig. 5:**
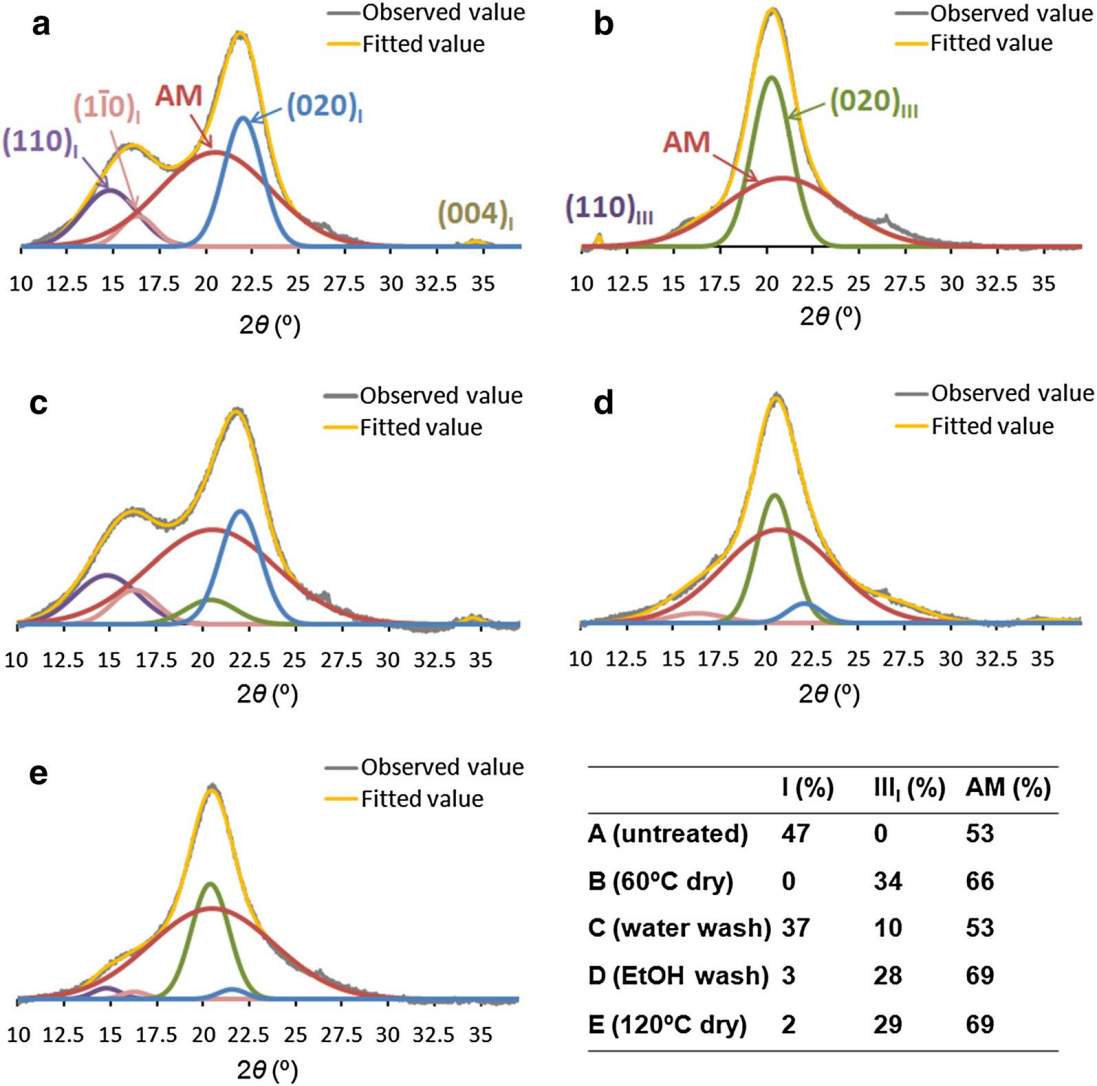
X-ray diffraction profiles. **a** untreated CS; **b** EDA-soaked CS followed by drying at 60 °C; **c** EDA-soaked CS followed by water-washing and air-drying; **d** EDA-soaked CS followed by ethanol-washing and air-drying; **e** EDA-soaked CS followed by drying at 120 °C. *AM* amorphous cellulose

Deconvolution of X-ray diffractogram was applied to figure out the transformation of allomorphic forms with various EDA separation methods (Fig. 5). The fitted curves were highly matched to measured curves (*R*^2^ was greater than 0.99 for each sample). It was observed that cellulose of untreated CS was composed of cellulose I with characteristic peak (110), (1Ī0), and (020) at Bragg angle of around 14.8°, 16.3°, and 22.2°, respectively. Amorphous cellulose peak was much broader with maximum intensity around 21°. The untreated cellulose contained 47 % cellulose I and 53 % amorphous cellulose (crystallinity index, CI, is 0.47) calculated by peak areas. Cellulose allomorph after EDA soaking and air-dry process was cellulose III_I_ with characteristic peak (110) and (020) at 11.3° and 20.3°, respectively. Cellulose III_I_ accounted for 20 % in this cellulose and another 80 % was amorphous cellulose (CI = 0.2). However, cellulose allomorph after pretreatments was a mixture of cellulose I and cellulose III_I_ in many cases. Cellulose of EDA-soaked CS followed by water-washing contained 37 % cellulose I and 10 % cellulose III_I_. During the transformation of EDA-soaked cellulose to cellulose III_I_, 3 and 2 % cellulose I was still present for ethanol-dry and oven-dry process, respectively.

The content of cellulose allomorphs was also obviously influenced by pretreatment temperature and EDA loading (Table 1). With EDA loading increasing, content of cellulose III_I_ and amorphous cellulose increased, and content of cellulose I decreased. These tendencies were observed for pretreated CS at 120, 150, and 180 °C. Furthermore, with pretreatment temperature increasing from 20 to 100 °C, content of amorphous cellulose decreased and content of cellulose III_I_ increased, which is agreed with previous report [23]. When temperature was higher than 100 °C, content of cellulose I began to increase while content of cellulose III_I_ decreased. The same results were also observed using Avicel as the substrate (Additional file 1: Table S3).

**Table 1:** Crystalline cellulose and amorphous cellulose contents of pretreated CS at different pretreatment conditions

Pretreatment temparature (°C)	EDA loading (mL/g biomass)	Cellulose I (%)	Cellulose III (%)	Amorphous cellulose (%)
120	0.2	32	15	53
120	0.4	27	20	53
120	0.6	25	22	53
120	0.8	15	23	62
120	1	2	29	69
20	1	5	20	75
40	1	3	32	65
60	1	0	34	66
80	1	0	35	65
100	1	0	37	63
120	0.6	25	22	53
150	0.6	28	14	58
180	0.6	33	13	54

### Morphological changes of the pretreated CS

**Fig. 6:**
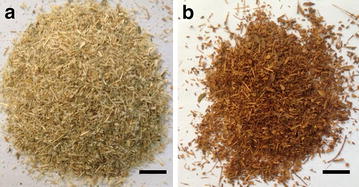
Untreated CS (**a**) and pretreated CS (**b**). EDA loading: 1.0 mL/g biomass; temperature: 120 °C; closed reaction for 20 min and then open drying for 20 min. *Scale bars* 1 cm

Stereoscope images of untreated and pretreated CS are shown in Fig. 6. The pretreated CS became dark brown compared to untreated CS. No other visible change in morphology was observed.

**Fig. 7:**
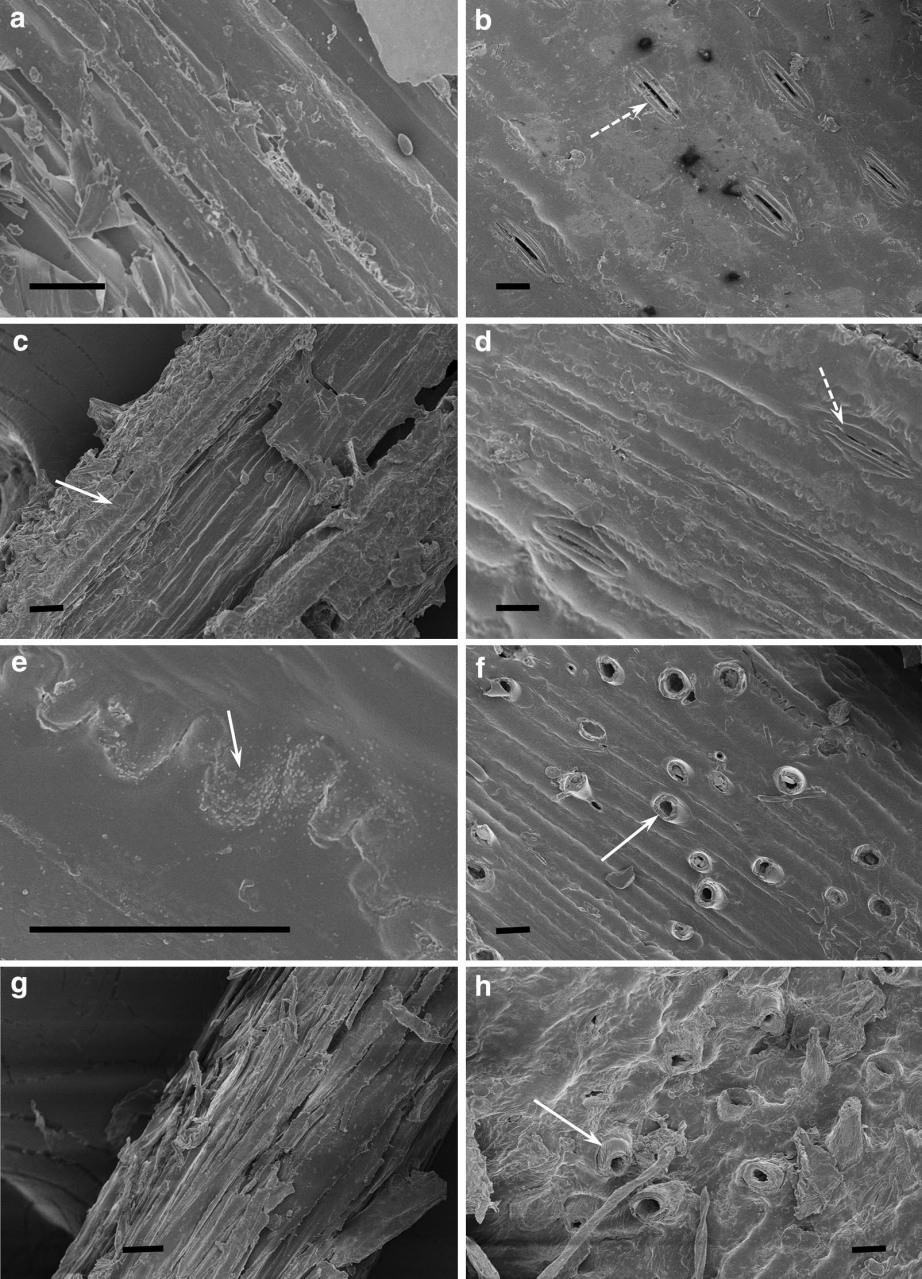
SEM images of untreated CS (**a**, **b**), pretreated CS (**c**, **d**, **e**, **f**) and pretreated CS followed by water-washing (**g**, **h**). *Scale bars* 20 µm

The representative images from scanning electron microscopy (SEM) and transmission electron microscopy (TEM) were selected from more than ten images of each sample. The microstructures of pretreated CS on SEM images exhibited significant differences from the untreated CS (Fig. 7). The surface of untreated stem (Fig. 7a) and leaf (Fig. 7b) was smooth. In contrast, the surfaces of EDA-pretreated stem (Fig. 7c) and leaf (Fig. 7d) were rough with convex configuration and corrugated appearance (arrow in Fig. 7c, e). The similar morphology was reported for AFEX pretreatment [24]. It was interesting that volcano-like holes were found at some pieces of pretreated CS (arrow in Fig. 7f), which is different from stoma on the leaf (dotted arrow in Fig. 7b, d). The holes were probably formed by the gasification of EDA in the biomass during drying process. The holes were prone to occur at the thin and ductile cell walls, such as corn husk. The pretreated CS after washing revealed that the rough substance on surface was soluble and removable (Fig. 7g, h). In AFEX-pretreated biomass, this similar soluble substance was confirmed to be rich in lignin and hemicellulose [25, 26]. After washing, the fibrils were clear and slightly separated from each other. Besides the surface changes, no obvious changes of the particle size were observed.

**Fig. 8:**
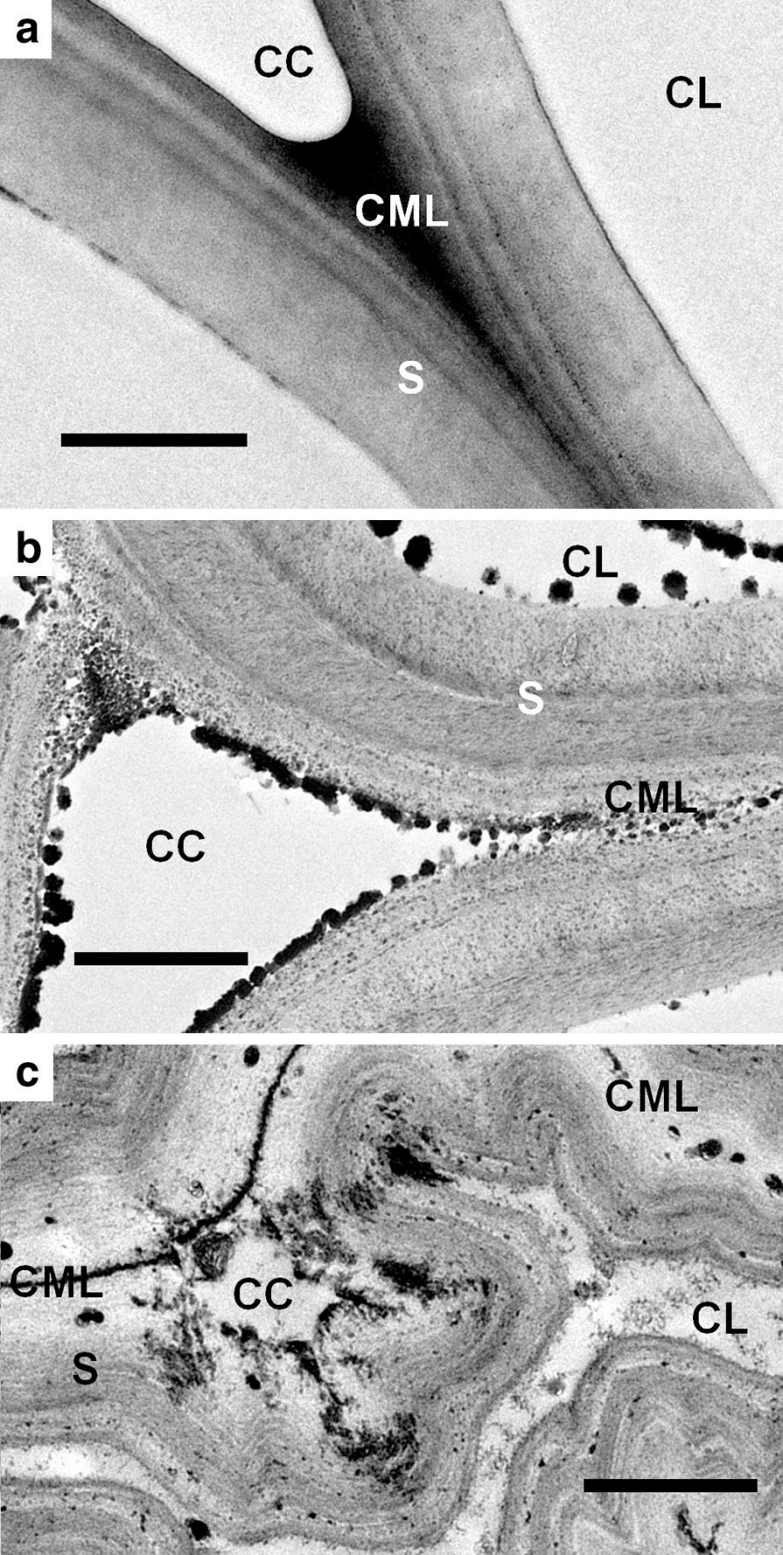
TEM images of untreated CS (**a**) and pretreated CS at 120 °C (**b**) and 150 °C (**c**). *CC* cell corners, *CL* cell lumen, *CML* compound middle lamella, *S* secondary wall layers. *Scale bars* 1 µm

TEM images further showed the cell wall changes during EDA pretreatment (Fig. 8). Untreated cell walls displayed relatively uniform staining pattern across the cell wall layers, indicating the fine distribution of lignin in cell corners, compound middle lamella, and secondary wall layers (Fig. 8a). The cell walls of the pretreated samples showed the dramatic evidence for lignin re-localization. For the cell walls after pretreatment at 120 °C, coalesced lignin-rich globules (up to hundreds of nanometers in diameter) were extruded outwards from the secondary wall layers and deposited on the cell wall surface (Fig. 8b), which is consistent with the SEM images. The lignified globules with similar size have been reported for dilute acid pretreated CS [27]. Cell walls after pretreatment at 150 °C showed kinking and curling (bending deflection) secondary walls and delamination pores in the secondary wall layers (Fig. 8c). The coalesced lignin on the cell wall surfaces presented not spherical but core–corona structure (one theory is lignin hydrophobic core and polysaccharide hydrophilic corona) [28], which is similar to the structure of AFEX and liquid hot water pretreatment [25, 28].

### NMR analysis

**Fig. 9:**
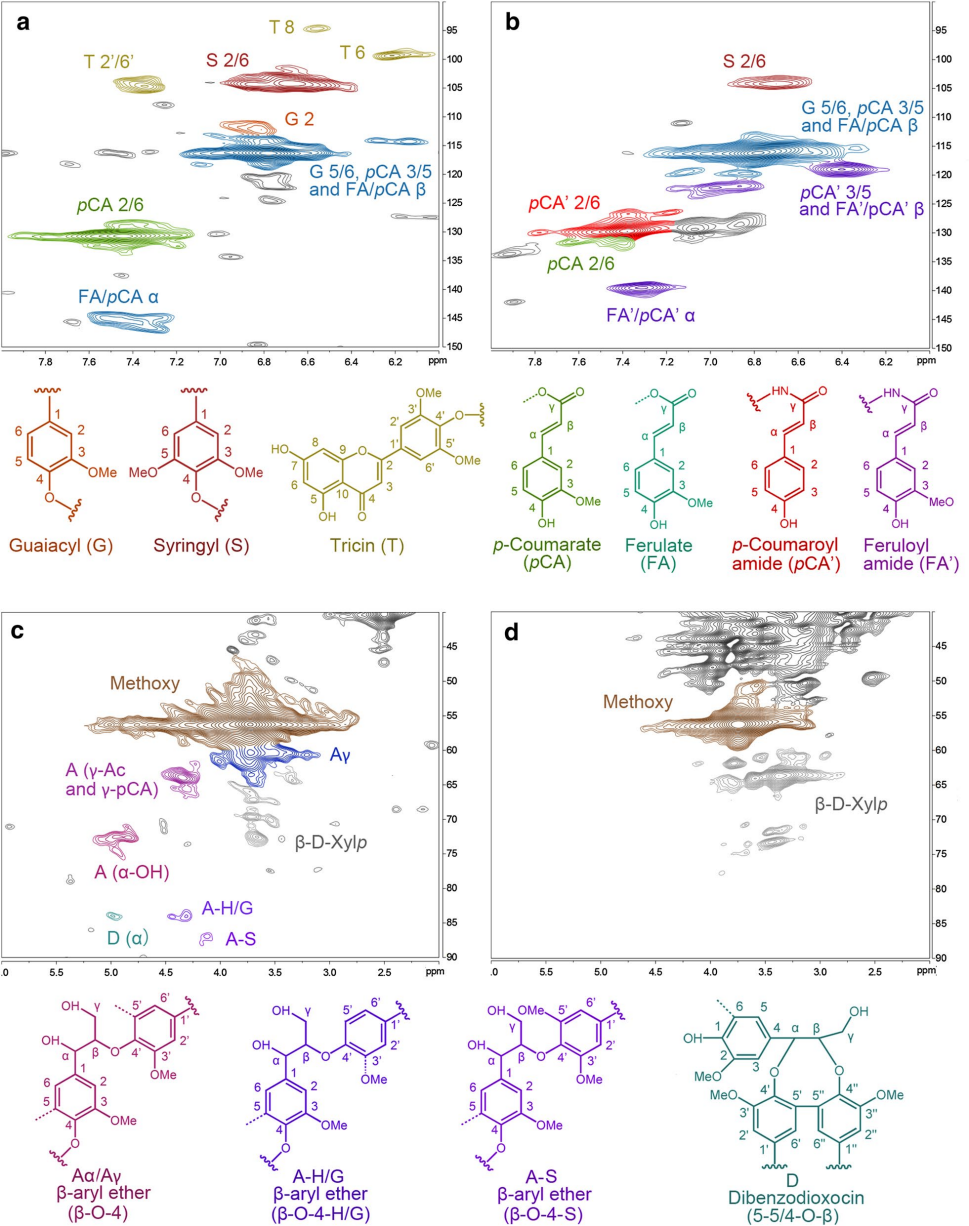
HSQC NMR spectra. Aromatic regions of DL isolated from untreated CS (**a**) and EDA-pretreated CS (**b**) and aliphatic regions of DL isolated from untreated CS (**c**) and EDA-pretreated CS (**d**)

In order to characterize the changes of lignin, main aromatic groups (*δ*_C_/*δ*_H_ 150–90/8.0–6.0, Fig. 9a, c) and inter-unit structures (*δ*_C_/*δ*_H_ 90–50/6.0–2.0, Fig. 9b, d) of isolated dioxane lignin (DL) were determined by 2D HSQC NMR. Chemical shift assignments of various lignin moieties were referred to previous studies [29–33]. In the aromatic region, DL isolated from untreated CS (Fig. 9a) was constituted by guaiacyl unit (G), syringyl unit (S), tricin (T), *p*-coumarate (*p*CA), and ferulate (FA), which was consistent with typical features of most herbaceous lignin. The cross peaks of *δ*_C_/*δ*_H_ 103.9/6.7 and *δ*_C_/*δ*_H_ 110.8/6.97 were assigned to ^13^C-^1^H correlations of S 2/6, G 2. Signals from T were identified at *δ*_C_/*δ*_H_ 94.1/6.56 (T8), *δ*_C_/*δ*_H_ 98.8/6.22 (T6), *δ*_C_/*δ*_H_ 104.04/7.30 (T 2’/6’). Several ^13^C-^1^H correlations of *p*CA were identified at *δ*_C_/*δ*_H_ 130.0/7.47 (*p*CA 2/6), *δ*_C_/*δ*_H_ 115.5/6.79 (*p*CA 3/5), *δ*_C_/*δ*_H_ 144.7/7.47 (*p*CA *α*). For EDA-pretreated CS (Fig. 9b), T structure and most *p*CA/FA structures were diminished. *p*-Coumaroyl amide (*p*CA′) and feruloyl amide (FA′), which are derived from *p*CA and FA, respectively, were observed at *δ*_C_/*δ*_H_ 129.2/7.47 and *δ*_C_/*δ*_H_ 139.7/7.47. The inter-units of lignin mainly composing of *β*-O-4 were found in the aliphatic regions of the HSQC spectrum of untreated CS (Fig. 9c). The signals of *δ*_C_/*δ*_H_ 71.8/4.8, *δ*_C_/*δ*_H_ 82–88/4.5–3.9, and *δ*_C_/*δ*_H_ 59.4/3.4 were ascribed to the correlation of a position of A *α*-OH, *β* position of A-H/G/S, and A*γ* in *β*-O-4 structure. The contours around *δ*_C_/*δ*_H_ 65-62/4.5-4 were assigned to the acylation at *γ*-OH by *p*CA and acetate. Trace amount of correlation signals from dibenzodioxocin (D) was detected at *δ*_C_/*δ*_H_ 83.4/4.9. In pretreated CS, the signals attributed to *β*-O-4 and 5–5′ structures were diminished (Fig. 9d), implying the linkages between the lignin monomers were cleaved and the lignin molecular weight was decreased.

### Ethanol fermentation and mass balance

**Fig. 10:**
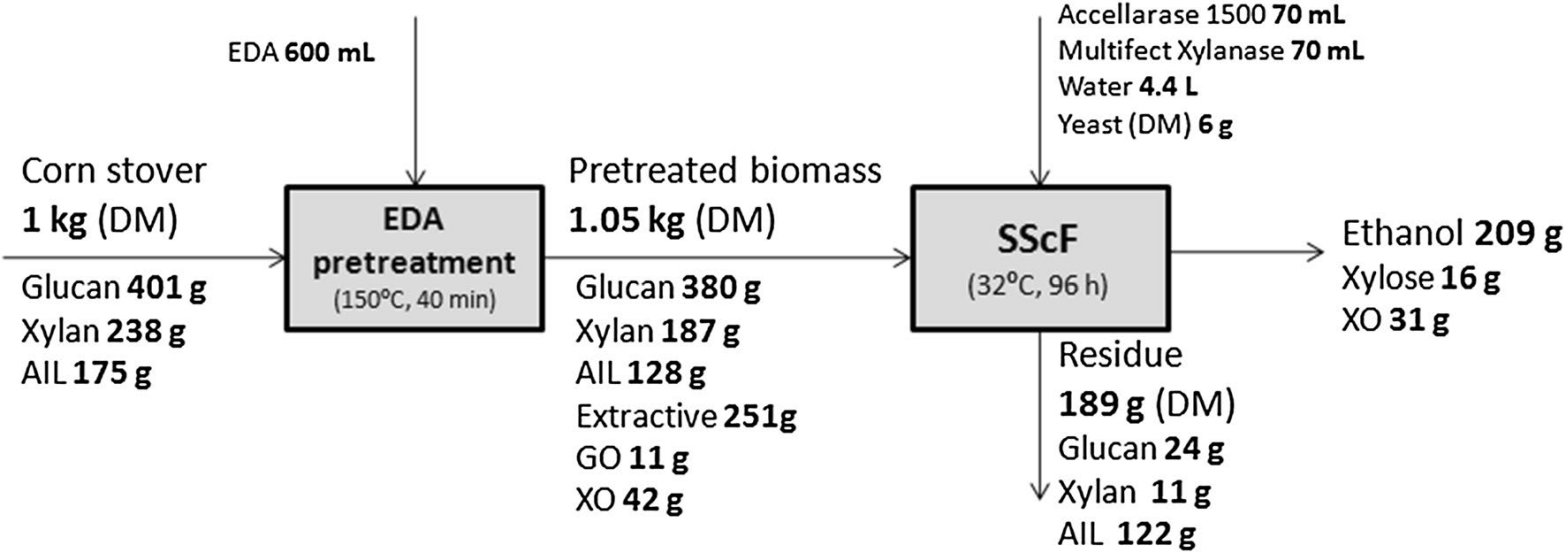
Mass balance from raw CS to ethanol by EDA pretreatment and SScF process. SScF was conducted at 6 % glucan loading. *DM* dry matter, *AIL* acid-insoluble lignin, *GO* gluco-oligomer, *XO* xylo-oligomer

The pretreated CS was subjected to simultaneous saccharification and co-fermentation (SScF) (Fig. 10). Surprisingly, EDA accelerated glucose and xylose utilization in ethanol fermentation at EDA concentration of 1-10 g/L (Additional file 1: Figure S1). At 96 h in SScF, 209 g ethanol was produced based on 1 kg dry matter of untreated CS. 12 g glucan and 7 g xylan was lost after pretreatment. 24 g glucan and 11 g xylan remained in the residue and 47 g xylose in hydrolysate (including oligomer) was not utilized after SScF process. Ethanol metabolic yield (based on hydrolyzed glucan and xylan) and ethanol yield (based on glucan and xylan in untreated CS) were 77 and 57 %, respectively.

## Discussion

As expected, EDA converted cellulose I to cellulose III_I_ except using water-washing (Fig. 5), which agreed with previous report [17]. Cellulose I was increased as pretreatment temperature increasing (Table 1). EDA–cellulose I complex gradually returned to cellulose I when heating temperature above 130 °C. Our findings further demonstrated that EDA loading and temperature also affected the content of cellulose III_I_ and amorphous cellulose (Table 1). During EDA pretreatment, low EDA loading (<1 mL/g biomass) changed cellulose I to cellulose III_I_ incompletely. According to previous research, a monoclinic unit cell of cellulose I-EDA crystal is composed of one anhydrous glucose residue and one EDA molecule [16], and only about 0.1 mL EDA/g biomass was incorporate into cellulose per g CS. However, the practical loading we used was much higher than the expected loading. This may be determined by the thermodynamic equilibrium between cellulose and EDA. The similar loading was used for AFEX pretreatment: about 1 g ammonia per g biomass, in which lower ammonia loading resulted in a decreased cellulose conversion as well [5]. Besides allomorph transformation, EDA pretreatment significantly reduced CI, which was also benefit to cellulose digestibility (Table 1, Additional file 1: Figure S2). EDA molecules preferentially penetrate the hydrophilic edges of the stacked sheets and enlarge cellulose III_I_ volume in their (010) direction [15] and thus increase the accessibility to water. It was reported that the enzymatic saccharification rate of cellulose III_I_ was about 5 times higher than cellulose I [19]. However, other studies found that initial rates of digestion were strongly correlated with amorphous content, not the allomorph type [23, 34]. In this study, the cellulose with more amorphous cellulose exhibited much higher digestibility than cellulose III (Table 1, Additional file 1: Figure S2). Moreover, cellulose III exhibited an increased digestibility compared to cellulose I (glucan conversions for cellulose I and cellulose III after 72 h enzymatic hydrolysis were 57 and 90 %, respectively) (Additional file 1: Figure S3). Our results agreed with previous studies [34] that digestibility of cellulose allomorph decreased as following: amorphous cellulose >cellulose III >cellulose I.

Although EDA changed cellulose allomorph and CI, delignification was another important factor impacting the cellulose digestibility. As pretreatment temperature increasing, glucose yield of pretreated Avicel was decreased as its CI increased (Additional file 1: Figure S2), while glucose yield of pretreated CS increased. The increase of glucose yield of CS is mainly attributed to the AIL content reduction, although CI of pretreated CS still increased as temperature increased (Additional file 1: Figure S2). Previous study showed that lignin plays a more important role than cellulose crystallinity on the digestibility of lignocellulose [35]. Both chemistry and physical barrier of lignin characteristics lead to the inhibition of enzymatic hydrolysis of lignin [36]. Lignin delocalization was found in EDA-pretreated CS (Figs. 7, 8). In EDA pretreatment at low temperature (under 120 °C), lignin coalesced to globules in and on the cell walls (Fig. 8b). At higher temperature, more lignin and hemicellulose coalesced and extruded from secondary walls and middle lamella, resulting in kinking and delamination of cell walls with broken holes (Fig. 8c). The sizes of the holes are similar to cellulase and facilitate the accessibility of cellulase [25]. Besides, EDA pretreatment converted 10–50 % of lignin to acid-soluble lignin (Figs. 3, 4). The ammonolysis of *p*-coumarate esters and the cleavage of *β*-O-4 ethers were the main reactions in EDA pretreatment (Fig. 9), which reduced the lignin molecular weight and increased lignin solubility. The cleavage of ester bonds in FA revealed that the cross-linkage of lignin–carbohydrate complex (LCC) was removed (LCC was mainly linked by FA) [30], which facilitated the delocalization of lignin during EDA pretreatment. The ammonolysis of *p*-coumarate esters was also found in AFEX pretreatment [25]. About 20 mg ammonia/g biomass remained in biomass during AFEX pretreatment, in which about 8 mg ammonia ammonolyzed acetyl linkages, coumarate and ferulate esters to their corresponding amides [6, 37]. In addition, the ammoniation of lignin eliminated its inhibition to cellulase by preventing cross-linking of lignin–enzyme interactions [38].

**Fig. 11:**
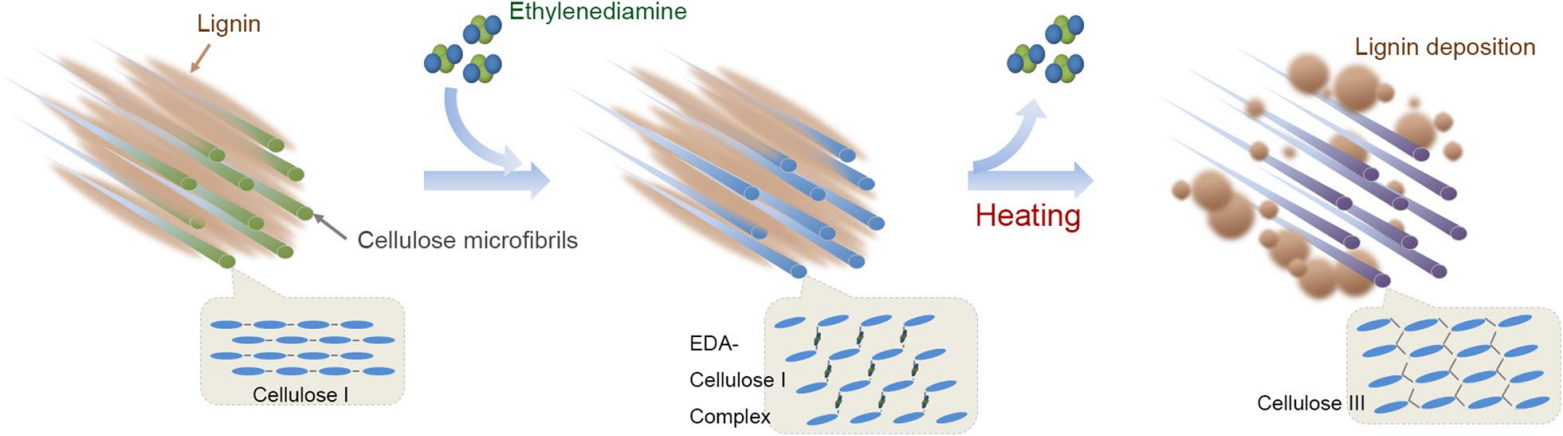
Schematic model for the effect of EDA pretreatment on lignocellulose. On the one hand, EDA with high temperature promotes lignin relocalization. On the other hand, EDA catalyzes cellulose I to cellulose III. Cellulose crystal lattice was designed based on figures from Wada [17] and Nishiyama [15]

Consequently, we supposed the process of EDA pretreatment (Fig. 11). With the addition of EDA, EDA molecules first penetrate into the cellulose crystal. With increasing temperature, EDA escape from the cellulose crystal and transform cellulose I to cellulose III_I_ (Table 1). At the same time, EDA ammonolyze esters into *p*-coumarate amides and cleave the ether linkages in lignin and the ester linkages in LCC (Fig. 9), which promotes lignin degradation and coalescence. With the escape of EDA, lignin extrudes outward and deposits on the surface of cell wall (Figs. 7, 8).

In this study, EDA pretreatment with a high solid-to-liquid ratio was elucidated to be effective on enhancing enzymatic hydrolysis of CS without washing, neutralization or detoxification. This pretreatment condition (EDA loading: 1 mL/g biomass, temperature: 120 °C) is similar to AFEX pretreatment conditions [6, 39]. EDA pretreatment has several favorable features including: most pentose is reserved in pretreated solid than acidic pretreatment, resulting in the effective use of pentose in the subsequent co-fermentation process; EDA is easily recovered and recycled, avoiding environment pollution. The dry pretreatment process (without water) appears to be more water saving. In addition, the pretreatment can be done by heating biomass at nearly atmospheric pressure.

EDA recycle will further improve the potential of the industrial application of EDA pretreatment. 2–9 g EDA remained in 100 g biomass after drying at high temperature above boiling point (Fig. 4), which will increase the cost of the pretreatment. Except for the consumption of EDA in *p*-coumarate amides and ferulate amides production, part of EDA remained in cellulose observed by IR spectra (Additional file 1: Figure S4). These noncovalent bound EDA could be separated theoretically by increasing drying time or increasing vacuum degree.

## Conclusions

The pretreatment of CS using EDA without high pressure was explored. EDA loading and the pretreatment temperature are the key factors for EDA pretreatment. The improvement of enzymatic digestibility is caused by the effect of EDA on the component of biomass, including: (1) the transformation of cellulose allomorph to cellulose III_I_ and amorphous cellulose from cellulose I_β_; (2) alteration of lignin chemical structure, morphological structure and location.

## Methods

### Materials

Corn stover (CS) was harvested in 2012 from the suburb of Tianjin, China. CS was washed, air-dried, and knife milled to pass through a 2-mm round screen and stored in supersacks in room temperature. The prepared CS contains 5 % moisture. The dry matter of CS composed of 40.1 % glucan, 23.8 % xylan, and 17.5 % AIL.

CSC and HFCS were prepared as control cellulosic substrates according to previous work [23]. In brief, CS was hydrolyzed with 0.6 % sulfuric acid at 130 °C for 1 h. HFCS was obtained after solid residue was washed exhaustively with distilled water. Further delignification was achieved by treating HFCS with acid chlorite to obtain CSC. HFCS was mixed with sodium chlorite (0.67 g/g biomass) and glacial acetic acid (1 mL/g biomass). The mixture was placed in water bath at 60 °C for 4 h with regular mixing. After acid chlorite delignification, the solid residue was water-washed till neutral. CSC and HFCS were air-dried until the moistures less than 10 %. Avicel PH-101 also as controlled cellulose was purchased from Sigma-Aldrich (MO, USA). EDA (>99 %) was purchased from Tianjin Yuanli Co., China.

Commercial cellulase Accellerase 1500^TM^ (89 mg/mL, 77 FPU/mL) and hemicellulase Multifect xylanase^TM^ (42 mg/mL) were gifted by Genencor (NY, USA).

### Pretreatment

**Fig. 12:**
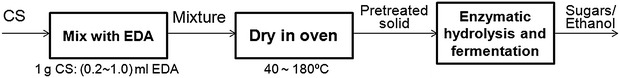
Schematic diagram for EDA pretreatment and refinery process

Schematic diagram of pretreatment process is shown in Fig. 12. Five grams (dry matter) of CS was mixed with pure EDA in glass plate (diameter = 12 cm). Aluminum foil was covered on the plate. The mixture was held in electric oven with designated temperature for 20 min. After this holding time, aluminum foil was taken off to evaporate EDA at the same temperature in the oven for another 20 min. The pretreated CS was achieved without any washing or neutralization steps and subjected to composition analysis, enzymatic hydrolysis, or simultaneously saccharification and co-fermentation.

### Chemical composition analysis

The compositions of untreated and pretreated CS were determined following the Laboratory Analytical Procedure (LAP) of the National Renewable Energy Laboratory (NREL). Glucose and xylose concentrations were determined by HPLC with Aminex HPX-87H column (Bio-rad, Hercules, CA, USA) at 60 °C with a mobile phase flow rate of 0.6 mL/min.

### X-ray diffraction (XRD)

The cellulose allomorph (CA) and crystalline index (CI) was measured using a D8 Fucos X-ray Diffractometer (Bruker AXS Co., Germany). Samples of particle size less than 125 µm were scanned at a speed of 4º/min, range from 2*θ* = 10 − 40º, and with a step size of 0.02° by positioning the samples on a quartz sample holder using Cu Kα radiation operated at 40 kV, 40 mA.

The deconvolution of the resulted diffractogram was performed using software PeakFit (SeaSolve Software Inc.). Gaussian functions were applied in curve fitting analysis.

Estimation of the content of cellulose I, cellulose III, and amorphous cellulose in the cellulosic samples was established by using the following equations:

1 $${\text{I\,\% = }}\frac{{{\text{A}}_{{\text{I}}} }}{{{\text{A}}_{{\text{I}}} {\text{ + A}}_{{{\text{III}}}} {\text{ + A}}_{{{\text{AM}}}} }} \times 100\,\%$$

2 $${\text{III\,\% = }}\frac{{{\text{A}}_{{{\text{III}}}} }}{{{\text{A}}_{{\text{I}}} {\text{ + A}}_{{{\text{III}}}} {\text{ + A}}_{{{\text{AM}}}} }} \times 100\,\%$$

3 $${\text{AM\,\% = }}\frac{{{\text{A}}_{{{\text{AM}}}} }}{{{\text{A}}_{{\text{I}}} {\text{ + A}}_{{{\text{III}}}} {\text{ + A}}_{{{\text{AM}}}} }} \times 100\,\%$$

where I %, III %, and AM % are the contents of cellulose I, cellulose III, and amorphous cellulose in samples, respectively; A_I_, A_III_,and A_AM_ are the peak areas of cellulose I, cellulose III, and amorphous cellulose, respectively.

The Bragg angles of peak (110), (1Ī0), (020), and (004) belonging to cellulose I are 14.8°, 16.3°, 22.3°, and 34.5°, respectively. The Bragg angles of peak (110) and (020) belonging to cellulose III are 11.3° and 20.0°, respectively. The Bragg angle of amorphous peak is around 20.5° [40, 41].

### Scanning electron micrograph (SEM)

SEM was carried out on a field emission scanning electron microscope (S4800, Hitachi, Co., Japan) after the samples were sputtered with a layer of gold.

### Transmission electron micrograph (TEM)

The methodology of sample preparation was referred to previous works [25, 42]. Samples were fixed in 2.5 % (w/v) glutaraldehyde in 0.2 M pH 7.2 sodium phosphate buffer twice for 6 min (2 min on, 2 min off, 2 min on) at room temperature followed by thoroughly washing with phosphate buffer. Dehydration of samples was achieved by transferring to vials containing a graded water–ethanol series (10 % steps for 30–90 % each of 15 min, 100 % for 30 min). After dehydration, the samples were infiltrated with LR White resin in increasing resin concentrations of 15, 30, 60, and 90 % resin diluted in ethanol and three times in 100 % resin. The resin-infiltrated samples were transferred to gelatin capsules and polymerized at 60 °C overnight. LR White-embedded samples were sectioned to 60 nm with a Diatome diamond knife on a Leica EM UC7 ultramicrotome (Wetzlar, Germany). Sections were collected on formvar-coated grids. Grids were post-stained for 10 min with 1 % aqueous uranyl acetate and 5 min with Reynolds lead citrate. Images were taken with a Gatan UltraScan 1000 camera (Gatan, Pleasanton, CA, USA) on an 80 kV JEM-1400 transmission electron microscope (JEOL, Japan).

### Lignin isolation and 2D NMR analysis

Lignin in untreated and pretreated CS was isolated following the procedure by Zeng [29, 30]. CS was milled in a planetary ball mill (DECO, China) with zirconium dioxide balls at 300 rpm for 4 h. The well-ground samples were extracted with ethanol for 12 h followed by repeated washings to remove extractives. The solids after extraction were air-dried, extracted by dioxane/water solution (96:4 v/v) for 48 h at room temperature, and air-dried. The dioxane soluble lignin (DL) from untreated CS was further purified by dissolving in an acetic acid/water solution (9:1 v/v) followed by the precipitation in cold deionized water and freeze-dried.

For each sample, 20 mg of DL was dissolved in 250 µL of 99.9 % DMSO-*d*_*6*_ with 0.05 % v/v TMS as internal standard. The heteronuclear single quantum coherence (HSQC) NMR spectra were recorded on a Varian Inova 400 MHz spectrometer (Agilent Technologies). The spectral width was 7211.5 Hz for ^1^H and 31,698 Hz for ^13^C. The number of scans was 32 with a 512 time increment. The relaxation delay was 0.43 s and the acquisition time was 0.07 s. Interactive integrations of the peaks in ^13^C/^1^H spectrum and contours in 2D HSQC plots were measured using MestReNova software.

### Enzymatic hydrolysis

Enzymatic hydrolysis was conducted with 20 ml reaction volume and 1 % glucan loading in 100 ml Erlenmeyer flasks. Accellerase 1500 and Multifect xylanase loading were 18 and 9 mg protein/g glucan, respectively. 50 mM citrate buffer (pH 4.8) with 50 mg/L ampicillin was used. Orbital incubator was set at 45 °C and 200 rpm. 0.5 mL aliquot was withdrawn during enzymatic hydrolysis and was centrifuged at 12,000 rpm for 5 min to separate hydrolysate from solid residue. Glucose and xylose concentrations in hydrolysates were determined by HPLC with Aminex HPX-87H column.

### Simultaneously saccharification and co-fermentation (SScF)

An engineered yeast *S. cerevisiae* SyBE005 with genetically constructed xylose utilizing pathway [43] was used in this study. Seed culture was prepared in YPX medium (10 g/L yeast extract, 20 g/L peptone and 20 g/L xylose) at 30 °C for 36 h. Inoculation size was OD_600_ = 4. Yeast and enzymes (enzymes used the same loading described above) were inoculated to pretreated CS medium with 6 % glucan loading at the same time. Ampicillin with finial concentration of 50 mg/L was used to prevent bacterial contamination. No other extra nutrient was added to the SScF medium. Experiment was carried out under anaerobic condition using shaking flasks in an orbital incubator at 32 °C and 150 rpm. To release the carbon dioxide produced at early stage, a syringe needle was pierced through the rubber stopper.

## Additional file

10.1186/s13068-015-0359-z **Table S1.** Compositions of pretreated CS from different EDA separation methods. **Table S2.** Compositions of pretreated CS from different temperatures and EDA loadings. **Table S3.** Crystalline cellulose (cellulose I and cellulose IIII) and amorphous cellulose contents of pretreated Avicel measured by XRD at different pretreatment conditions.** Figure S1.** Glucose (A), xylose (B), ethanol (C), and EDA (D) concentration during fermentation using *S.cerevisiae* SyBE005 with synthetic medium (20 g/L glucose, 10.5 g/L xylose, and different concentrations of EDA). **Figure S2.** Glucose yield in enzymatic hydrolysis of  pretreated CS (A), pretreated Avicel (B), acid insoluble lignin recovery of pretreated CS (C), and CI of pretreated Avicel (D) have the relationships to pretreatment temperature from 20 ºC to 100 ºC. **Figure S3.** Glucan conversion of untreated Avicel (cellulose I) and EDA pretreated Avicel at 120 ºC (cellulose III) in enzymatic hydrolysis. Enzymatic hydrolysis was conducted with 20 mL reaction volume and 1 % glucan loading in 100 mL Erlenmeyer flasks. Accellerase 1500 loading was 18 mg protein/g glucan. 50 mM citrate buffer (pH 4.8) with 50 mg/L Ampicillin was used. Orbital incubator was set at 45 ºC and 200 rpm. **Figure S4.** FTIR spectra of untreated and EDA pretreated CS and Avicel. Pretreatment temperature was 120 ºC. EDA loading was 1 mL/g biomass. Drying time was 40 min. Peaks at 1630 cm^−1^ of EDA-CS and EDA-Avicel stand for C-N vibration in ethylenediamine, indicating ethylenediamine remain in pretreated CS and Avicel.

## Abbreviations

EDA: ethylenediamine
CS: corn stover
CSC: corn stover cellulose
HFCS: hemicellulose-free corn stover
AIL: acid-insoluble lignin
DL: dioxane lignin
SScF: simultaneous saccharification and co-fermentation
AFEX: ammonia fiber expansion
CI: crystalline index
AM: amorphous cellulose
HPLC: high performance liquid chromatography
XRD: X-ray diffraction
HSQC NMR: heteronuclear single quantum coherence nuclear magnetic resonance
SEM: scanning electron microscope
TEM: transmission electron microscope
IR: infrared radiation
H: hydroxyphenyl unit
G: guaiacyl unit
S: syringyl unit
T: trincin
*p*CA: *p*-coumaric acid
FA: ferulic acid
*p*CA’: *p*-coumarate amide
FA’: ferulate amide
G: glucan
GO: gluco-oligomer
X: xylan
XO: xylo-oligomer
CC: cell corners
CL: cell lumen
CML: compound middle lamella
S: secondary wall layers
LCC: lignin–carbohydrate complex
FPU: filter paper unit
