# Supplementary material for: Ethylenediamine pretreatment changes cellulose allomorph and lignin structure of lignocellulose at ambient pressure
Source: Biotechnol Biofuels. 2015 Oct 29;8:174. doi: 10.1186/s13068-015-0359-z (PMC4625619; doi:10.1186/s13068-015-0359-z)
Supplement: Supplementary file 1 — 10.1186/s13068-015-0359-z Table S1. Compositions of pretreated CS from different EDA separation methods. Table S2. Compositions of pretreated CS from different temperatures and EDA loadings. Table S3. Crystalline cellulose (cellulose I and cellulose IIII) and amorphous cellulose contents of pretreated Avicel measured by XRD at different pretreatment conditions. Figure S1. Glucose (A), xylose (B), ethanol (C), and EDA (D) concentration during fermentation using S.cerevisiae SyBE005 with synthetic medium (20 g/L glucose, 10.5 g/L xylose, and different concentrations of EDA). Figure S2. Glucose yield in enzymatic hydrolysis of pretreated CS (A), pretreated Avicel (B), acid insoluble lignin recovery of pretreated CS (C), and CI of pretreated Avicel (D) have the relationships to pretreatment temperature from 20 ºC to 100 ºC. Figure S3. Glucan conversion of untreated Avicel (cellulose I) and EDA pretreated Avicel at 120 ºC (cellulose III) in enzymatic hydrolysis. Enzymatic hydrolysis was conducted with 20 mL reaction volume and 1 % glucan loading in 100 mL Erlenmeyer flasks. Accellerase 1500 loading was 18 mg protein/g glucan. 50 mM citrate buffer (pH 4.8) with 50 mg/L Ampicillin was used. Orbital incubator was set at 45 ºC and 200 rpm. Figure S4. FTIR spectra of untreated and EDA pretreated CS and Avicel. Pretreatment temperature was 120 ºC. EDA loading was 1 mL/g biomass. Drying time was 40 min. Peaks at 1630 cm−1 of EDA-CS and EDA-Avicel stand for C-N vibration in ethylenediamine, indicating ethylenediamine remain in pretreated CS and Avicel. [file 13068_2015_359_MOESM1_ESM.docx]

Table S1 Compositions of pretreated CS from different EDA separation methods.

| EDA separation | Glucan | Xylan | Acid-insoluble lignin |
| --- | --- | --- | --- |
| Air dry | 35.2% | 20.1% | 14.0% |
| Water wash | 42.6% | 24.3% | 16.2% |
| Ethanol wash | 36.4% | 21.5% | 14.1% |
| Oven dry | 35.2% | 17.0% | 7.6% |

Table S2 Compositions of pretreated CS from different temperatures and EDA loadings.

| Temperature (ºC) / EDA loading (ml/g biomass) | Glucan | Xylan | Acid-insoluble lignin |
| --- | --- | --- | --- |
| 120/0.2 | 36.7% (1.4%) | 21.1% (0.8%) | 15.9% (0.0%) |
| 120/0.4 | 36.3% (0.0%) | 19.4% (0.2%) | 13.1% (0.1%) |
| 120/0.6 | 35.9% (1.0%) | 18.1% (0.5%) | 10.8% (0.3%) |
| 120/0.8 | 34.7% (0.6%) | 17.3% (0.1%) | 9.3% (0.5%) |
| 120/1.0 | 35.2% (0.7%) | 17.0% (0.3%) | 7.6% (0.3%) |
| 150/0.2 | 37.2% (0.7%) | 19.9% (0.4%) | 14.8% (0.4%) |
| 150/0.4 | 35.7% (0.1%) | 17.8% (0.2%) | 12.1% (0.2%) |
| 150/0.6 | 35.4% (0.4%) | 17.5% (0.1%) | 12.0% (0.7%) |
| 180/0.2 | 36.1% (0.6%) | 20.0% (0.5%) | 15.0% (0.9%) |
| 180/0.4 | 37.9% (0.4%) | 18.9% (0.3%) | 14.0% (0.7%) |
| 180/0.6 | 36.7% (0.6%) | 18.3% (0.4%) | 14.2% (0.3%) |

Table S3 Crystalline cellulose (cellulose I and cellulose III_I_) and amorphous cellulose contents of pretreated Avicel measured by XRD at different pretreatment conditions.

| Pretreatment conditions | Cellulose I (%) | Cellulose III (%) | Amorphous cellulose (%) |
| --- | --- | --- | --- |
| Untreated | 56% | 11% | 33% |
| 20ºC dry | 6% | 11% | 83% |
| 40ºC dry | 1% | 24% | 75% |
| 60ºC dry | 1% | 40% | 59% |
| 80ºC dry | 2% | 39% | 59% |
| 100ºC dry | 0% | 33% | 67% |
| 120ºC dry | 0% | 35% | 65% |
| Water wash and air dry | 33% | 21% | 46% |
| Ethanol wash and air dry | 7% | 28% | 65% |


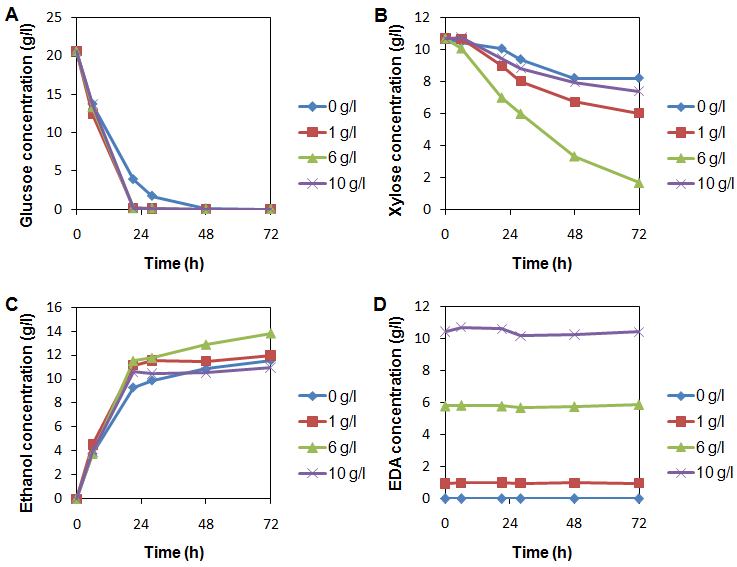


Figure S1 Glucose (A), xylose (B), ethanol (C), and EDA (D) concentration during fermentation using *S.cerevisiae* SyBE005 with synthetic medium (20 g/l glucose, 10.5 g/l xylose, and different concentrations of EDA).


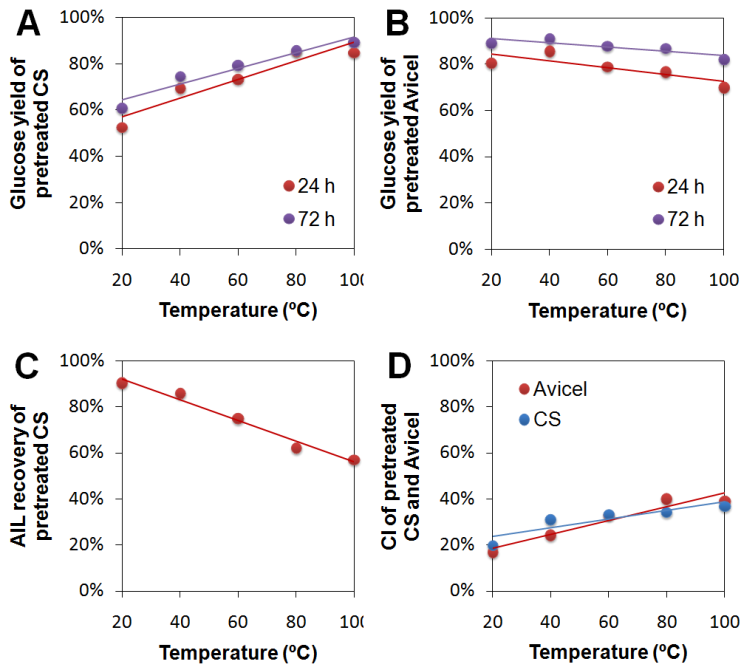


Figure S2 Glucose yield in enzymatic hydrolysis of pretreated CS (A), pretreated Avicel (B), acid insoluble lignin recovery of pretreated CS (C), and CI of pretreated Avicel (D) have the relationships to pretreatment temperature from 20ºC to 100ºC.


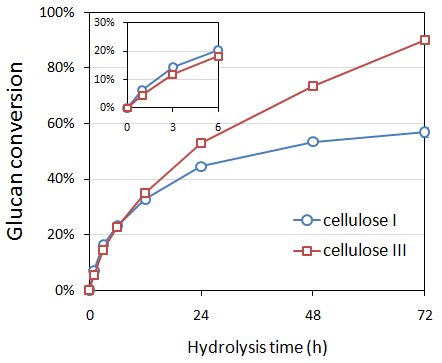


Figure S3 Glucan conversion of untreated Avicel (cellulose I) and EDA pretreated Avicel at 120ºC (cellulose III) in enzymatic hydrolysis. Enzymatic hydrolysis was conducted with 20 ml reaction volume and 1% glucan loading in 100 ml Erlenmeyer flasks. Accellerase 1500 loading was 18 mg protein/g glucan. 50 mM citrate buffer (pH 4.8) with 50 mg/L Ampicillin was used. Orbital incubator was set at 45ºC and 200 rpm.


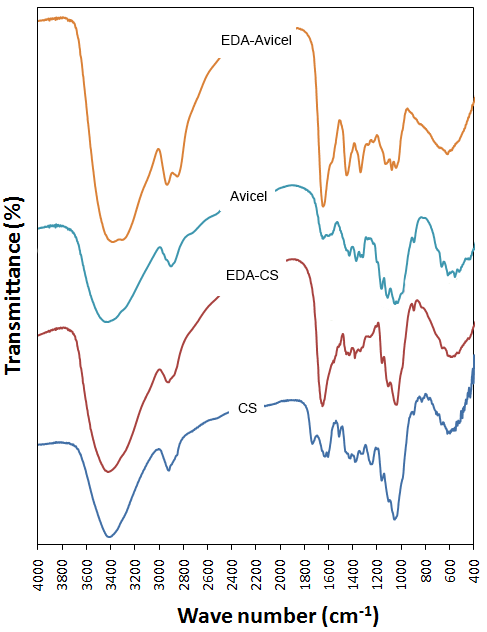


Figure S4 FTIR spectra of untreated and EDA pretreated CS and Avicel. Pretreatment temperature was 120ºC. EDA loading was 1 mL/g biomass. Drying time was 40 min. Peaks at 1630 cm^-1^ of EDA-CS and EDA-Avicel stand for C-N vibration in ethylenediamine, indicating ethylenediamine remain in pretreated CS and Avicel.
